# Genome assembly forensics: finding the elusive mis-assembly

**DOI:** 10.1186/gb-2008-9-3-r55

**Published:** 2008-03-14

**Authors:** Adam M Phillippy, Michael C Schatz, Mihai Pop

**Affiliations:** 1 Center for Bioinformatics and Computational Biology, University of Maryland, College Park, MD 20742, USA

## Abstract

We present the first collection of tools aimed at automated genome assembly validation. This work formalizes several mechanisms for detecting mis-assemblies, and describes their implementation in our automated validation pipeline, called *amosvalidate*. We demonstrate the application of our pipeline in both bacterial and eukaryotic genome assemblies, and highlight several assembly errors in both draft and finished genomes. The software described is compatible with common assembly formats and is released, open-source, at http://amos.sourceforge.net.

## Abstract 2

A collection of software tools is combined for the first time in an automated pipeline for detecting large-scale genome assembly errors and for validating genome assemblies.

## Rationale

Sequence assembly errors exist in both draft and finished genomes. Since the initial 'draft' sequence of the human genome was released in 2001 [1,2], great effort has been spent validating and finishing the official sequence. During this process, it became clear that the original draft sequences were not entirely accurate reconstructions of the genome [3-6]. It was also reported in 2004 that 'finished' human bacterial artificial chromosome (BAC) sequences contained a single base-pair error per every 73 Kbp of sequence and more significant mis-assemblies every 2.6 Mbp [3]. Some errors had left large stretches of sequence omitted, rearranged, or otherwise deformed. After five more years, the human genome is nearly complete; however, validation and finishing has been a largely manual, and expensive, process requiring additional laboratory work and sequencing.

For many other genomes, cost prohibits manual sequence validation, and the genomes are often left as draft assemblies. Such sequences likely contain many errors, and recent calls for caution have been made regarding assembly quality [7]. Too often, assembly quality is judged only by contig size, with larger contigs being preferred. However, large contigs can be the result of haphazard assembly and are not a good measure of quality. It has been difficult to gauge assembly quality by other means, because no automated validation tools exist.

The following sections describe a software pipeline for validating the output of assembly programs. To begin, we provide an overview of the genome assembly process and catalog the signatures (inconsistencies) that result from an incorrect reconstruction of the genome. We then describe the methods and software tools we have developed to identify such signatures, and provide examples of their use in several recent genome projects.

### Double-barreled shotgun assembly

**Figure 1:**
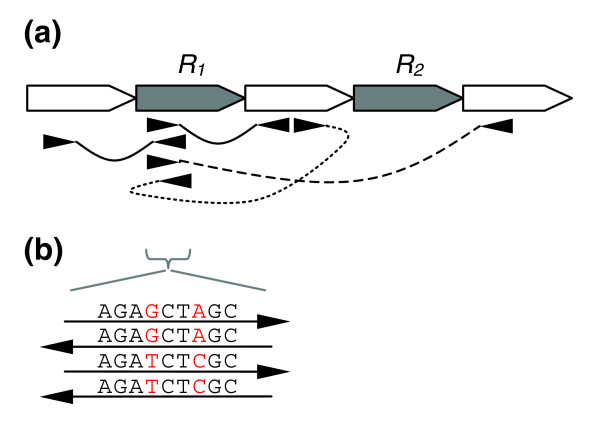
Misplaced reads caused by the two copy repeat *R *and leading to **(a) **unsatisfied mate-pairs and **(b) **correlated SNPs. Unique sequence is shown in white and repetitive sequence in gray. Example mate-pairs are drawn as connected arrow heads. Properly oriented mates point towards each other, and properly sized pairs are connected with a solid line. All mates can be satisfied and the correlated SNP removed if the bottom two reads in *R*_1_ are moved to *R*_*2*_.

Shotgun sequencing, the most widely used DNA sequencing technique to date, involves three major steps: first, the DNA is randomly sheared into fragments (shotgun step); second, the ends of each fragment are sequenced, resulting in two reads per fragment (double-barreled sequencing step); and third, the original DNA sequence is reconstructed from the reads (assembly step). Newly emerging sequencing technologies also follow this general model, albeit with different strategies for each step. The first two steps are highly automated, although the assembly step remains a difficult challenge for any sequencing technology. Assembly would be a trivial process if each read had a unique placement; however, all but the simplest organisms contain duplicated sequences (repeats) throughout their genome. These repeats confuse the assembly process, since reads originating from distinct copies of the repeat appear identical to the assembler. Additionally, for near-identical repeats, it is difficult to differentiate sequencing error from the polymorphism between repeat copies. This may cause an assembler to incorrectly place repetitive reads, resulting in mis-assembly. The pairing of reads sequenced from opposite ends of a same DNA fragment (mate-pairs, or paired ends) helps to disambiguate read placements within and around repeats, as show in Figure 1a where ambiguous placements can be resolved by reads whose mates are anchored in unique sequence.

In a correct assembly, the layout of the reads, and implicitly, the layout of the original DNA fragments, must be consistent with the characteristics of the shotgun sequencing process used to generate the data. In general, a correct assembly must satisfy the following constraints. First, the sequences of overlapping reads must agree; exceptions are sequencing errors, polyploid organisms, and the assembly of mixed samples such as non-clonal or out-bred organisms. Second, the distance between mated reads must be consistent with the size of the fragments generated from the random shearing process; exceptions are chimeric DNA fragments. Third, mated reads must be oriented towards each other, that is, they must come from opposite strands of the sequenced DNA; exceptions are chimeric DNA fragments, and alternative pairing methods (for example, transposon libraries). Fourth, the placement of reads throughout the assembly must be consistent with a random shearing process, represented mathematically as a Poisson process [8]; exceptions are cloning or sequencing biases. Fifth, all reads provided to the assembler must be consistent with the resulting assembly, that is, every read must perfectly match at least one location in the reconstructed genome; exceptions are sequencing errors, incomplete trimming of the sequencing vector, and the presence of contaminants.

All five of these constraints are subject to some degree of inaccuracy, as indicated by the exceptions indicated above. A single violation is, therefore, not usually conclusive of mis-assembly. Instead, multiple, coinciding constraint violations need to be observed in order to infer the presence of an error in assembly. The following section describes the primary types of mis-assemblies and the pattern of constraint violations they exhibit.

### Mis-assembly signatures

The majority of mis-assemblies fall into two generalized categories: repeat collapse and expansion; and sequence rearrangement and inversion. Each type has distinct mechanisms for mis-assembly and results in different signatures. The first type of mis-assembly results from incorrectly gauging the number of repeat copies in a genome and including too few or too many copies. Differences in copy numbers of certain repeats are known to cause phenotypic differences between organisms (for example, Huntington's disease [9]); therefore, a correct assembly of such regions is essential. The second type of mis-assembly results from shuffling the order of multiple repeat copies, thereby rearranging the unique sequence in between. This type of mis-assembly, if uncaught, could be misinterpreted as a biological rearrangement event. There is a chance such false conclusions have already been drawn due to mis-assembled genomes, and, therefore, the mechanisms and signatures of these mis-assemblies need to be examined in more detail.

In both collapse and rearrangement events, reads may be placed in the wrong copy of a repeat. Small differences between repeat copies, often single nucleotide polymorphisms (SNPs) caused by mutations that arose in the different copies independently, are useful indicators of collapsed or otherwise mis-assembled repeats. While disagreements due to sequencing errors tend to occur at random, the differences caused by mis-assemblies can be identified by their correlated location across multiple reads (Figure 1b). Some correlated SNPs may also occur due to heterogeneous sequencing samples or sequence-specific lab errors, and, therefore, correlated SNPs by themselves are not always sufficient evidence of mis-assembly.

### Repeat collapse and expansion

**Figure 2:**
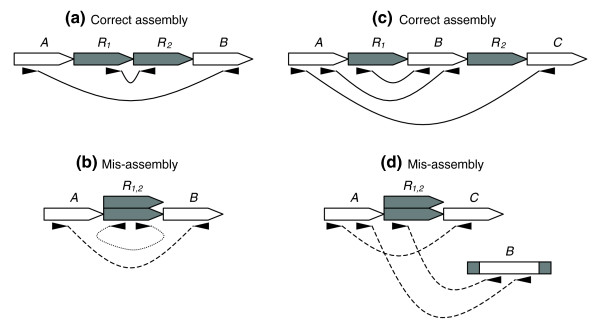
Mate-pair signatures for collapse style mis-assemblies. **(a) **Two copy tandem repeat *R *shown with properly sized and oriented mate-pairs. **(b) **Collapsed tandem repeat shown with compressed and mis-oriented mate-pairs. **(c) **Two copy repeat *R*, bounding unique sequence *B*, shown with properly sized and oriented mate-pairs. **(d) **Collapsed repeat shown with compressed and mis-linked mate-pairs.

In the case of a repeat collapse, the assembler incorrectly joins reads originating from distinct repeat copies into a single unit (Figure 2). The opposite occurs in an expansion, where extra copies of a repeat are included in the assembly. These often result in a greater (or lesser) density of reads than is expected from the random shotgun process. A missing repeat copy causes reads to 'pile up' in the remaining copies, thereby increasing read density. For example, in a genome sampled at 8-fold coverage with reads of 800 bp in length, the reads are expected to be placed at approximately 100 bp increments throughout the genome. The collapse of a two copy repeat results in an even denser packing of the reads in the single remaining copy - within the collapsed repeat the reads are spaced by roughly 50 bp and the depth of coverage (number of reads spanning a specific location) is increased to about 16-fold. The reverse is true for an expansion mis-assembly, where the read density drops below normal coverage.

In the case where two repeat copies are adjacent to each other, that is, a tandem repeat, the reads that span the boundary between the two copies cannot be placed in the collapsed assembly. These reads only partially align to the assembly and exhibit an identifiable mis-assembly signature where they appear to wrap-around the boundary of the repeat. In addition, mate-pairs spanning the boundary between the two copies, but internal to the tandem, also appear to wrap around and mates spanning the tandem are shorter than expected (Figure 2b). For expansions, spanning mates appear stretched. When two repeat copies are separated by a unique region, a collapse forces the intervening section of DNA out of the assembly, leading to the creation of two separate contigs. Any mate-pairs that were spanning one of the repeat copies now link from the excised contig to the middle of the collapsed contig (Figure 2d). An insertion results in a similar signature, with mates spanning the insertion boundary linking to separate contigs. In general, any non-overlapping placement of two contigs with respect to each other results in the violation of mate-pair constraints, indicating the presence of a mis-assembly.

### Rearrangements and inversions

**Figure 3:**
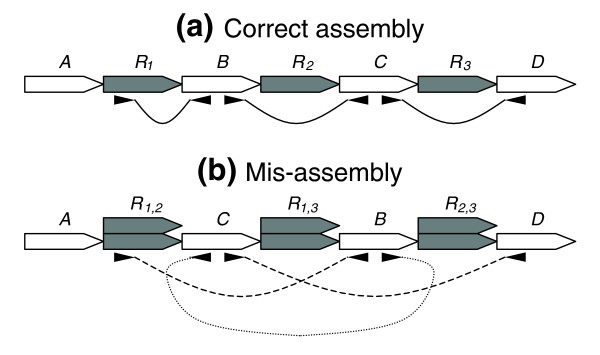
Mate-pair signatures for rearrangement style mis-assemblies. **(a) **Three copy repeat *R*, with interspersed unique sequences *B *and *C*, shown with properly sized and oriented mates. **(b) **Mis-assembled repeat shown with mis-oriented and expanded mate-pairs. The mis-assembly is caused by co-assembled reads from different repeat copies, illustrated by the stacked repeat blocks.

**Figure 4:**
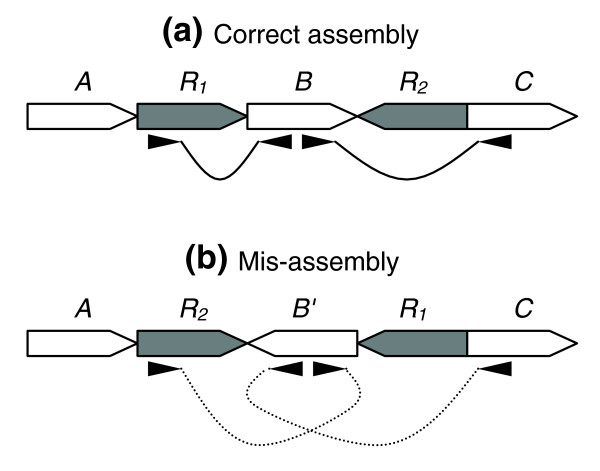
Mate-pair signatures for inversion style mis-assemblies. **(a) **Two copy, inverted repeat *R*, bounding unique sequence *B*, shown with properly sized and oriented mate-pairs. **(b) **Mis-assembled repeat shown with mis-oriented mate-pairs.

Even when an assembler correctly gauges the number of repeat copies, thereby avoiding the situations described above, mis-assemblies are still possible. Such a situation is shown in Figure 3, where, by incorrectly redistributing reads between the three copies of repeat *R*, the regions *B *and *C *of the genome have been swapped. Inversions are a special case of rearrangement, occurring when two repeat copies are oriented in opposite directions, thereby allowing the intervening region to be inverted (Figure 4). These 'inverted' repeats can easily confuse the assembler, and can also result in genomic rearrangements *in vivo*, such as those detected within the plasmids of *Bacillus anthracis *Ames [10]. In the case of mis-assembly, heterogeneities may result within the mis-assembled repeat copies, due to mis-placed reads, unless the repeat copies are identical. In addition, mate-pair constraints are violated for any mate-pairs spanning the repeat unit. If the repeat is not spanned by mate-pairs, this class of mis-assembly is harder to detect, and it is sometimes possible to mis-assemble the genome without violating a single mate-pair constraint. While a random placement of the reads among repeat copies would result in violations, assembly programs often place the reads such that the constraints are satisfied, thereby obscuring the mis-assembly.

### Prior work

Gene Myers' original formulation of the assembly problem stated that an assembly of a genome must match (in terms of the Kolmogorov-Smirnoff test statistic) the statistical characteristics of the process used to generate the data [11]. To our knowledge, this is the first formulation of the assembly problem that explicitly takes into account the presence of repeats in genomes. Furthermore, this formulation provides a theoretical framework for developing assembly validation tools. A simple version of this approach, the arrival-rate statistic (A-statistic), is used within Celera Assembler to identify collapsed repeats [12].

The validation of genome assemblies was originally done manually, in conjunction with genome finishing efforts aimed at generating the complete sequence of organisms. Validation software was generally provided as an add-on to assembly editors like Consed [13], Staden package [14], or TIGR Editor (in-house software used at The Institute for Genomic Research). New interest in developing tools for assessing the quality of assemblies was spurred by the race to finish the human genome, in particular by the competition between the publicly led effort [1] and the private challenger Celera Genomics [2]. The ensuing controversy and flurry of papers comparing the two assemblies underscored the absence of objective and reliable tools for assembly validation. Eventually, the human assemblies were verified through comparisons to a collection of independently generated data such as finished BAC clones [15], gene content [16,17], and (at a lower resolution) genomic physical maps [1,2,18].

Such comparative validation methods have limited applicability. First, they rely on the availability of a 'gold standard' provided by independently generated and often manually curated data. Second, these methods can only detect mis-assemblies covered by the sparse curated data. A more general approach utilizes just the assembly data themselves, such as the constraints imposed by the mate-pairs, whose placement within the assembly must be consistent with the characteristics of the shotgun process. For example, a visual display of mate-pairs, the clone-middle-plot, was used to compare the two different assemblies of the human genome [19], and the popular assembly viewer/editor Consed [13] includes the means to explore the placement of paired reads along the genome as a tool for identifying mis-assemblies. Our own assembly viewer, Hawkeye [20], presents the assembly as a tiling of paired reads, and provides several visualization options aimed at highlighting possible assembly problems. An integrated analysis of mate-pairs is built into the quality control module of the Arachne assembler [21,22]. The Arachne approach detects clusters of unsatisfied mate-pairs and low quality bases to estimate the probability of mis-assembly for each region of the assembly. In addition, two standalone programs are available for mate-pair based evaluations: BACCardI [23] allows the user to visualize the placement of mate-pairs along the genome and highlights those mate-pairs that are incorrectly placed with respect to each other, and TAMPA [24] uses a computational geometry algorithm to identify clusters of mis-mated reads that are characteristic of a mis-assembly.

Despite its many benefits, mate-pair based validation may produce many false positives due to the inherent inaccuracy in the experimental protocols. For example, in a correct assembly many mate-pairs would be characterized as incorrect, specifically those representing the tails of the mate-pair size distribution. This problem can be alleviated using statistical hypothesis testing, an approach used by the compression-expansion (CE) statistic [25]. In short, for every position in the genome, the CE statistic represents the deviation - in number of standard errors - of the observed mean mate-pair size from the mean size of the shotgun library (the statistical Z-test). A CE value near 0 indicates the local distribution of sizes is in agreement with the global distribution, while large (for example, greater than 3) negative (positive) values indicate the presence of a compression (expansion) in the assembly. This statistic is less sensitive to the variance of mate-pair sizes, and, therefore, much more sensitive in identifying true errors.

An alternative approach to mis-assembly detection and resolution is taken by DNPTrapper [26]. This tool focuses on the heterogeneities between co-assembled reads to detect collapsed repeats, and provides an interface for manually separating the individual copies, using the Defined Nucleotide Position framework of Tammi *et al. *[27]. Another sequence based approach introduced by Kim *et al*. [28] examines the distribution of sequences within all reads to identify repetitive, and therefore difficult to assemble, regions.

Despite their utility, none of the tools described above take into account more than one measure of assembly correctness. The Methods section describes *amosvalidate*, the first integrated pipeline for assembly validation that combines multiple observations and validation techniques to more accurately detect mis-assemblies. This comprehensive approach increases the sensitivity and specificity of mis-assembly detection, and focuses validation on the most probable mis-assemblies. Regions identified as mis-assembled are output in AMOS message format, thereby enabling the integration with other validation pipelines, as well as manual inspection with the Hawkeye assembly visualization tool.

## Methods

Violations of the five basic rules described in the Rationale are most commonly caused not by mis-assemblies, but by statistical variation or errors in the underlying data provided to the assembler. The high-throughput biochemical processes used to sequence genomes are error-prone, leading to non-random coverage across the genome, sequencing errors, and mis-paired reads. Furthermore, experimental measurements (for example, mate-pair sizes) are inherently noisy. Separating such experimental artifacts from errors introduced by mis-assemblies is one of the main requirements of a robust validation pipeline. To reduce the effect of these errors on the analysis, multiple sources of evidence must be combined to increase the specificity of mis-assembly detection. In addition, certain types of mis-assembly can only be detected by specific methods, while the sequencing strategy employed may restrict the types of information that can be used for validation (for example, many emerging sequencing technologies do not yet generate mate-pair information). In the remainder of this section we describe our approach for assembly validation based on several measures of assembly consistency. We will describe the types of mis-assemblies detected by each of the measures and conclude with examples of how these measures are integrated to reveal potential assembly errors.

### Mate-pair validation

The mate-pair validation component of the pipeline separately identifies the four types of mis-mated reads: mates too close to each other; mates too far from each other; mates with the same orientation; and mates pointing away from each other. Reads with mates not present in the assembly or whose mates are present in a different contig are also reported. In order to reduce the impact of noise in the underlying data, multiple mate-pair violations must co-occur at a specific location in the assembly before reporting the presence of an error. In addition, the CE statistic described in the Rationale aids in the identification of clusters of compressed or expanded mate-pairs.

The actual size of shotgun libraries is sometimes mis-estimated by sequencing centers; therefore, a mechanism to re-estimate the library parameters on the basis of mate-pairs that are co-assembled within a contig is required. Reads that occur too close to the end of a contig may bias the distribution in favor of short mate-pairs (the mate-pairs at the upper end of the distribution would fall beyond the end of the contig and, therefore, not contribute to the calculations) and are thus ignored. Specifically, we ignore every read that is closer than *μ *+ 3*σ *from the end of the contig when re-estimating the parameters of a library with mean *μ *and standard deviation *σ*. It is often necessary to iterate this process a few times until convergence. The size of a library is re-estimated only if the size of a sufficient number of mate-pairs can be estimated and only if either the mean or the standard deviation change significantly from the original estimate.

In addition to mate-pair violations, regions of inadequate depth of coverage are identified, as well as regions that are not spanned by any valid mate-pair (that is, 0X fragment coverage). The latter may represent situations where non-adjacent regions of the genome were co-assembled across a repeat. When computing fragment coverage we exclude from consideration the paired reads sequenced from each fragment. This is necessary in order to make the distinction between read and fragment coverage at a specific location. By our definition, the read coverage cannot drop below one within a contig, but the fragment coverage can be as low as zero, indicating the absence of long-range support for this region of the contig. At the typical depths of read coverage used in sequencing, each location in the genome is generally well covered by mate-pairs.

### Repeat analysis

Most mis-assemblies are caused by repeats; therefore, understanding the repeat structure of a genome can aid in the validation of its assembly. Some repeats can be found by aligning the assembled contigs against each other and identifying duplicated regions. Tools like Vmatch [29] and Tandem Repeat Finder [30] can be used for the *de novo *identification of repetitive regions in the assembly, which can then be examined for correctness. This approach, however, is not appropriate for all types of mis-assemblies. For example, the complete collapse of a two copy tandem repeat into a single copy cannot be detected by comparative means.

For validation purposes we are not simply interested in identifying the location of all repeats, rather we are trying to identify those repeats that have been assembled incorrectly, in particular those repeats that cannot be easily identified through comparative analysis. Specifically, we try to identify regions of the genome that are over-represented in the set of reads, yet appear unique when examining the consensus sequence generated by the assembler. We achieve this by comparing the frequencies of *k*-mers (*k*-length words) computed within the set of reads (*K*_*R*_) with those computed solely on the basis of the consensus sequence (*K*_*C*_). *K*_*R *_is the frequency of all *k*-mers inside the clear range of all reads; and *K*_*C *_is the frequency of all *k*-mers across the consensus sequence of the assembled contigs. The forward and reverse complements of each *k*-mer are combined into a single frequency. The normalized *k*-mer frequency, K* = *K*_*R*_*/K*_*C*_, is computed for each *k*-mer in the consensus, where a deviation from the expected K* (in a correctly assembled region, K* should approximately equal the average depth of coverage *c*) reveals those repeats likely to be mis-assembled. For example, *K*_*R *_measured across a two copy repeat is 2*c *regardless of whether the assembly is correct or not. If the repeat is correctly assembled into two distinct copies, *K*_*C *_= 2, and, therefore, K* = *c*. If instead the repeat is collapsed, then *K*_*C *_= 1 and K* = 2*c*, indicating the presence of a mis-assembly. This approach is particularly powerful when used in conjunction with the technique described below for identifying dense clusters of SNPs because the two methods are complementary. SNP based detection will find collapsed, heterogeneous repeats, while K* will reveal collapsed, identical repeats.

### Coverage analysis

As described in the introduction, the collapse of a repeat results in an increase in the depth of coverage. This characteristic signature can, therefore, be used to detect the presence of mis-assemblies. For short repeats with low copy number (for example, two-copy repeats), this effect cannot be distinguished from the variation in coverage caused by the randomness of the shotgun sequencing process, limiting the applicability of this method to repeats that occur in many copies throughout the genome, or to relatively long stretches of repetitive DNA (sustained deviations from the average depth of coverage are unlikely to occur by chance). The significance of observing a certain level of over-representation, given the parameters of the shotgun process, can be calculated through statistical means (see the A-statistic used by Celera Assembler [12]).

### Identification of micro-heterogeneities

Under the assumption of a random distribution of sequencing errors, and an independent random sampling of the genome during the shotgun process, it is unlikely that any two overlapping reads have sequencing errors at the same consensus position. While there are several examples of sequence-dependent sequencing errors that invalidate our assumption of independence between errors occurring in different reads (for example, hard-stops caused by the formation of DNA hair-pin structures, or long homopolymer regions characterized by frequent polymerase slippage), these assumptions are true for the vast majority of sequencing errors. Also, the following discussion assumes the genome being sequenced represents a single clonal organism. The assembly of non-clonal bacterial populations or heterozygous eukaryotes is characterized by frequent heterogeneities between co-assembled reads. Such situations are often known *a priori *and the validation pipeline can be adjusted accordingly.

As described in the introduction, mis-assemblies often result in the presence of micro-heterogeneities (SNPs) that are correlated across multiple overlapping reads. Identifying such polymorphisms can, therefore, indicate potential errors in the assembly. To identify mis-assembly induced SNPs, and distinguish them from simple sequencing errors, we take advantage of the base quality values provided by the sequencing software. The *phred *quality values [31], for example, represent the log-probability of error at every base in the sequence. Under the assumption of independence of errors across reads, we can sum these values to estimate the probability of observing multiple correlated errors at a specific location in the assembly, and mark as polymorphism those locations where this probability exceeds a specific threshold. For example, the probability of error for two reads reporting the same base, each with a quality value of 20, is equivalent to the probability of error for a single base with a quality value of 40 (*P*(error) = 1/10,000). This is, in essence, the same approach used by genome assembly software in assigning quality values for the consensus sequence [32]. For each heterogeneous column of the multi-alignment, reads are grouped into 'alleles' by which nucleotide they report. The quality values for each read in an allele are summed, and if two or more alleles have a quality value of 40 or greater (by default), the difference is marked as a SNP. For a concrete example, if two reads report a *C *each with quality 25, and three reads report a *G *each with quality 20, the qualities of the alleles are 50 and 60, respectively, and the difference is marked as a *C/G *SNP. If, however, the quality of either allele is below 40, the difference is not marked as a SNP. In addition, our software evaluates the proximity of SNPs to further increase the confidence in our predictions; clusters of SNPs that occur within a small range in the assembly are likely indicative of a mis-assembly. By default we mark regions containing at least 2 high quality SNPs occurring within a 500 bp window.

Note that this technique for mis-assembly detection can also be applied in heterogeneous genomes, for example, by identifying regions with a significantly higher SNP density than the background rate. In such genomes, however, we expect much higher false-positive rates due to localized regions of heterogeneity, requiring this method to be combined with other validation measures.

### Read breakpoint analysis

The reads provided to an assembler must be consistent with the resulting assembly. Thus, examining how the un-assembled reads (also called singletons, or shrapnel) disagree with the assembly can reveal potential mis-assemblies. To compare un-assembled reads to a consensus we use the *nucmer *component of the MUMmer package [33,34], and allow fragmented alignments to the consensus. For instance, a mapping that aligns the first half of a read to a different region than the second half, but at 100% identity, is preferable to a mapping that aligns the read contiguously at 80% identity. The fragmented, high identity alignment is more likely because the read sequence should be nearly identical to the consensus sequence, modulo sequencing errors. From among all alignments of a read to the genome we choose the placement that maximizes the sum of *len(A*_*i*_*)* * idy(A_*i*_*)* over all alignment segments *A*_*i*_, where *len(A*_*i*_*)* and *idy(A*_*i*_*)* are the length and percent identity of the *i*^*th *^segment of alignment *A*, and *len(A*_*i*_*)* is adjusted where necessary to avoid scoring the overlap between adjacent segments twice. This scoring function estimates the number of non-redundant bases matching the consensus, and the MUMmer utility *delta-filter *computes an optimal alignment using this function and a modified version of the Longest Increasing Subsequence (LIS) algorithm [35]. Most mappings consist of a single alignment that covers the entire read, while the fragmented mappings indicate either incorrect trimming of the read or the presence of a mis-assembly.

For fragmented alignments, the locations where the alignment breaks - boundaries of alignment fragments that do not coincide with the ends of the read - are called 'breakpoints'. Under the assumption that all reads map perfectly to the assembly, breakpoints indicate the presence of errors, either in the assembly, or in the reads themselves (for example, incomplete trimming, or chimeric fragments). Breakpoints supported by a single read are rarely cause for concern, and can often be explained by errors in the reads themselves. However, multiple reads that share a common breakpoint often indicate assembly problems. These multiply supported breakpoints are identified, after the alignment process described in the previous section, by sorting the boundaries of fragmented alignments by their location in the consensus, and reporting those that occur in multiple reads. In addition, for each read we store a vector of coordinates encoding all breakpoints in the alignment of the read to the genome. This vector allows us to determine not only if two reads share common breakpoints, but also if they have similar mappings to the consensus. For each breakpoint, we then examine the cluster of reads with similar alignment signatures to characterize different classes of mis-assemblies in much the same way mate-pairs are used to characterize collapse, inversion, and so on. But while mate-pair and coverage methods can only bound a mis-assembly to a certain region, breakpoints can identify the precise position in the consensus at which the error occurs.

### Integration of validation signatures

Our validation pipeline, *amosvalidate*, executes the analyses described above to tag regions that appear mis-assembled. Independently, each analysis method may report many false-positives that reflect violations of the data constraints, but that do not necessarily represent mis-assemblies or incorrect consensus sequence. A common example is clusters of overlapping stretched or compressed mate-pairs caused by a wide variance in fragment sizes rather than mis-assembly. By combining multiple mis-assembly signatures we increase the likelihood that the tagged regions identify true errors in the assembly. For example, a region with a largely negative CE value is more likely to indicate the presence of a collapsed repeat if an unusually high density of correlated SNPs is also present. This particular combination is especially strong, since mate-pair and sequence data are independent sources.

Since some types of signatures do not necessarily tag the exact location of a mis-assembly, combining mis-assembly signatures requires considering not only overlapping signatures, but also those that occur in close proximity. To combine mis-assembly signatures, the pipeline identifies regions in the assembly where multiple signatures co-occur within a small window (2 Kbp by default). If multiple signatures of at least two different evidence types occur within this window, the region is flagged as 'suspicious'. Each such region is reported along with detailed information about the individual signatures, and forms the initial focus for subsequent validation and correction efforts. For manual analysis, these regions, along with the individual mis-assembly features, can be viewed alongside the assembly data in the AMOS assembly viewer, Hawkeye.

### Implementation details

The validation modules of *amosvalidate *are implemented in C++ and included as part of the AMOS assembly package [36]. AMOS is a modular, open-source framework for genome assembly research and development, which provides integration between software modules through a centralized data store and a well defined API. This framework allows developers to focus on a particular area of interest, for example, scaffolding, without needing to develop a complete assembly infrastructure. Furthermore, AMOS can import data from common assembly programs and formats - ACE, NCBI Assembly/Trace Archives [37], Arachne [38,39], Celera Assembler [12], PCAP [40], Phrap [41], Phusion [42] and Newbler [43], allowing for the integration of AMOS modules into existing assembly pipelines.

## Results

### Tandem repeat collapse in *B. anthracis*

**Figure 5:**
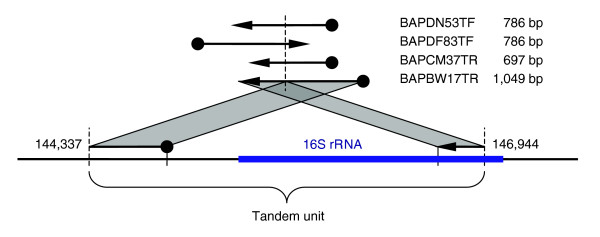
Breakpoint signature of mis-assembly in *B. anthracis *Ames Ancestor. The alignments of the four reads to the assembly indicate the collapse of a tandem repeat consisting of two copies of the section of the assembly between coordinates 144,337 and 146,944. Note how the alignment signature resembles the mate signature shown in Figure 2b.

The impetus for much of this work was a mis-assembly we detected in the parent strain of *B. anthracis *Ames Ancestor (RefSeq ID: NC_007530). As shown in Figure 5, an alignment breakpoint analysis detected four unassembled reads that only partially matched the assembly. The partial matches ended at the same locations in all reads, specifically at coordinates 144,337 and 146,944 in the assembled main chromosome of *B. anthracis*. This pattern is consistent with the collapse of a tandem repeat consisting of two copies of the sequence between these two coordinates. The four unassembled reads span the boundary between the two copies of the repeat, leading to the observed alignment in the incorrect assembly. Increased depth of coverage was also observed in the assembly, supporting the collapse hypothesis. This observation was confirmed by a close inspection of the assembly in this region, and the finishing team at TIGR was able to correct the assembly.

It is important to note that this genome had been finished at The Institute for Genomic Research (TIGR) and had already been deposited into GenBank at the time when this mis-assembly was identified. The mis-assembly had thus escaped detection despite the extremely stringent manual curation performed by the finishing teams at TIGR. Since finishing is primarily aimed at closing gaps, rather than fixing mis-assemblies, it is not that surprising that errors persist even in finished data. Examples like this reinforce recent calls for caution when dealing with all assemblies, not just those of draft quality [7].

### Example from *Drosophila virilis*

To test the scalability of *amosvalidate*, the pipeline was run on an assembly of the fruit fly *Drosophila virilis*. The genome was sequenced with the whole-genome shotgun method to approximately 8× coverage by Agencourt Bioscience Corporation, and assembled with both Celera Assembler and Arachne. The current best assembly, Comparative Analysis Freeze 1 (CAF1), is available from the consortium website [44] and comprises 13,530 scaffolds containing 18,402 contigs with a total length of approximately 189 Mbp. This assembly represents a reconciliation of both the Celera Assembler and Arachne results [25]. Because the read multi-alignment is not provided with the reconciled assembly, we describe the analysis of a small region of the Celera Assembler assembly. Due to the absence of a finished reference, it is impractical to evaluate our analysis on a larger scale.

**Figure 6:**
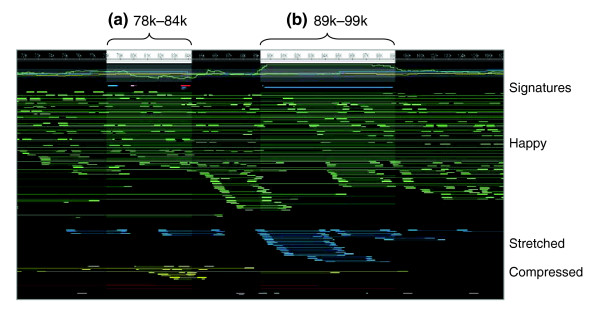
Hawkeye screen shot of an example *D. virilis *mis-assembly. Sequencing reads are represented as thick boxes connected to their mate by thin lines. Correctly sized (happy) mates are shown in green, stretched in blue, and compressed in yellow. A CE statistic plot is given at the top, with mis-assembly signatures plotted directly below as intervals. **(a) **The *amosvalidate *region, which appears to be a compression mis-assembly. **(b) **The *amosvalidate *region, which appears to be an expansion mis-assembly.

In a 556 Kbp contig of the Celera Assembler assembly, *amosvalidate *predicted 56 mis-assembly signatures and 6 suspicious regions. Two of the suspicious regions are at the extreme ends of the contig, and correctly identify the low quality sequence present at the ends of the contig. Two more regions are weakly supported by CE stretch and missing mate signatures, but do not appear to be egregious mis-assemblies. The remaining two regions, however, reflect obvious mis-assembly. The left-hand region (Figure 6a), positioned at 78,088-84,132, is supported by alignment breakpoint, missing mate, and correlated SNP signatures. In addition, the cluster of yellow, compressed mates at the bottom of Figure 6 correspond exactly with the position of the correlated SNPs. Examination of the multi-alignment at this position reveals two distinct sets of co-assembled reads. These lines of evidence together point to a collapse style mis-assembly. The right-hand region (Figure 6b), positioned at 89,408-98,979, is more subtle and supported only by CE expansion and SNP signatures. However, the overwhelming severity of the CE expansion caused by the cluster of blue, expanded mates at the bottom of Figure 6 suggest that additional sequence has been incorrectly inserted into this region.

The official, reconciled CAF1 assembly does not contain either of these mis-assemblies, independently confirming our analysis. Instead, the suspicious region is broken into multiple contigs, with the left half mapping to contig_16268 of the CAF1 assembly and the right half to contig_16269.

### Systematic evaluation of bacterial assemblies

To supplement the anecdotal results presented above, we have performed a systematic evaluation of assemblies using *amosvalidate*. Sequencing data for 16 bacterial genomes were collected and assembled with Phrap v0.990329 using the *phrap.manyreads *program with default parameters. Phrap was chosen because of its popularity, simplicity, and tendency to mis-assemble repetitive genomes. Similar experiments were attempted with Celera Assembler, but not enough mis-assemblies were produced to allow adequate validation. In larger genomes, Celera Assembler, and virtually all other assemblers, produce many errors; however, there are not enough fully finished eukaryotic genomes to allow comprehensive testing of our methods. For extensive and objective testing, bacteria were chosen as the assembly targets because many complete, finished genomes are available, thus providing a proper reference that can be used to identify true mis-assemblies.

The Phrap assemblies were aligned against the reference sequences using the MUMmer utility *dnadiff *to collect regions of mis-assembly. *dnadiff *performs a whole-genome alignment and compactly summarizes the location and characteristics of differences between two contig sets [45]. For aligning contigs to a reference genome, this process is identical to the read mapping discussed in the 'Read breakpoint analysis' section. Using the same algorithm, the contig set is mapped to the reference genome using *nucmer*, and the optimal mapping for each contig is identified. The alignment information is then parsed, and all alignment breakpoints are identified. By default, *nucmer *creates a contiguous alignment as long as the average nucleotide identity is greater than 70% for a 200 bp window; therefore, any stretch of greater than approximately 60 mis-matches will force the alignment to break. After alignment, the breakpoints are classified as insertions, deletions, rearrangements, or inversions based on their surrounding context. For example, a breakpoint between a forward-strand and negative-strand alignment on the same contig is classified as an inversion. For the Phrap contigs, only alignment differences that produced a breakpoint were considered as mis-assemblies. Small differences such as consensus SNPs, short indels (less than approximately 60 bp), and breakpoints occurring within the first 10 bp of a contig were ignored. All contigs less than 5,000 bp were also ignored because of their generally low quality.

*amosvalidate *was then run on all 16 Phrap assemblies to determine if the mis-assembled regions were correctly identified by our methods. Additional data file 1 lists the NCBI Taxonomy and RefSeq identifiers for the 16 reference genomes. Table 1 gives a summary of the Phrap induced mis-assemblies, along with statistics detailing the performance of *amosvalidate*. Table 2 gives specific details on the types of mis-assemblies introduced by Phrap, and the size characteristics of the *amosvalidate *features. Mis-joins (rearrangements) were the most prevalent type of mis-assembly reported by *dnadiff*.

**Table 1:** Accuracy of *amosvalidate *mis-assembly signatures and suspicious regions summarized for 16 bacterial genomes assembled with Phrap

				Mis-assembly signatures			Suspicious regions		
					
Species	Len	Ctgs	Errs	Num	Valid	Sens	Num	Valid	Sens
*B. anthracis*	5.2	87	2	1,336	21	100.0	127	2	100.0
*B. suis*	3.4	120	10	1,047	30	80.0	158	9	90.0
*C. burnetii*	2.0	55	22	1,375	70	100.0	124	19	100.0
*C. caviae*	1.4	270	12	625	16	83.3	50	8	66.7
*C. jejuni*	1.8	53	5	290	11	80.0	61	3	60.0
*D. ethenogenes*	1.8	632	12	688	22	91.7	88	9	100.0
*F. succinogenes*	4.0	455	21	1,670	27	95.2	266	14	66.7
*L. monocytogenes*	2.9	172	1	1,381	5	100.0	201	1	100.0
*M. capricolum*	1.0	17	3	83	0	0.0	16	0	0.0
*N. sennetsu*	0.9	16	0	91	0	NA	13	0	NA
*P. intermedia*	2.7	243	21	1,655	57	100.0	201	20	100.0
*P. syringae*	6.4	274	64	2,841	200	98.4	366	55	98.4
*S. agalactiae*	2.1	127	21	687	53	95.2	112	18	85.7
*S. aureus*	2.8	824	41	1,850	69	97.6	227	18	75.6
*W. pipientis*	3.3	2017	31	761	92	100.0	132	30	100.0
*X. oryzae*	5.0	50	151	2,569	379	100.0	100	69	100.0
									
Totals	46.8	5412	417	18,949	1,052	96.9	2,242	275	92.6

Species name, genome length (Len), number of assembled contigs (Ctgs), and alignment inferred mis-assemblies (Errs) are given in the first four columns. Number of mis-assembly signatures output by *amosvalidate *(Num) is given in column 5, along with the number of signatures coinciding with a known mis-assembly in column 6 (Valid), and percentage of known mis-assemblies identified by one or more signatures in column 7 (Sens). The same values are given in columns 8-10 for the suspicious regions output by *amosvalidate*. The suspicious regions represent at least two different, coinciding lines of evidence, whereas the signatures represent a single line of evidence. A signature or region is deemed 'validated' if its location interval overlaps a mis-assembled region identified by *dnadiff*. Thus, a single signature or region can identify multiple mis-assemblies, and *vice versa*, a single mis-assembly can be identified by multiple signatures or regions.

**Table 2:** Details on the types of mis-assemblies and feature characteristics for the results presented in Table 1

		Mis-assembly types				Mis-assembly signatures			Suspicious regions		
				
Species	Len	Ins	Del	Join	Inv	Num	aLen	%Len	Num	aLen	%Len
*B. anthracis*	5.2	0	0	2	0	1,336	831	21.5	127	5,546	13.6
*B. suis*	3.4	0	0	7	3	1,047	1,354	42.2	158	7,575	35.6
*C. burnetii*	2.0	0	0	13	9	1,375	1,106	74.3	124	11,455	69.4
*C. caviae*	1.4	0	0	11	1	625	320	14.1	50	3,896	13.7
*C. jejuni*	1.8	1	0	3	1	290	613	10.0	61	1,981	6.8
*D. ethenogenes*	1.8	0	0	8	4	688	691	26.5	88	4,116	20.2
*F. succinogenes*	4.0	0	1	19	1	1,670	1,387	57.5	266	7,396	48.8
*L. monocytogenes*	2.9	0	0	1	0	1,381	873	42.1	201	5,254	36.9
*M. capricolum*	1.0	3	0	0	0	83	835	6.8	16	3,005	4.7
*N. sennetsu*	0.9	0	0	0	0	91	512	5.4	13	2,328	3.5
*P. intermedia*	2.7	0	0	19	2	1,655	727	44.5	201	6,263	46.5
*P. syringae*	6.4	0	1	43	20	2,841	782	34.4	366	5,725	32.4
*S. agalactiae*	2.1	0	0	16	5	687	793	25.6	112	4,082	21.5
*S. aureus*	2.8	1	0	34	6	1,850	740	49.0	227	5,582	45.4
*W. pipientis*	3.3	0	0	17	14	761	1,206	28.1	132	6,395	25.8
*X. oryzae*	5.0	1	0	74	76	2,569	1,551	79.0	100	27,771	55.1
											
Totals	46.8	6	2	267	142	18,949	895	35.1	2242	6773	30.0

Phrap mis-assemblies are grouped into tandem insertion (Ins), tandem collapse (Del), mis-join (Join), and inversion (Inv) events in columns 3-6. Columns 7-9 give the total count (Num), average length (aLen), and total length as a percentage of genome (%Len) for the *amosvalidate *mis-assembly signatures. Columns 10-12 give the same information, but for *amosvalidate *suspicious regions.

In summary, the sensitivity of our methods is quite good; 96.9% of known mis-assemblies are identified by one or more *amosvalidate *signatures, and 92.6% are identified by one or more *amosvalidate *suspicious regions. However, the apparent specificity appears quite low. The over-prediction of mis-assembly signatures can be mostly ignored, because each signature represents a true violation of the five rules listed in the Rationale. These are meant to highlight inconsistencies in the assembly, and do not always correspond to actual mis-assemblies. The over-prediction of suspicious regions appears to indicate a limitation of our methods. In this case, it is mostly due to the nature of the Phrap algorithm. Because the version of Phrap used in our analysis disregards mate-pair information, many reads are placed in incorrect repeat copies. This leads to both correlated SNPs in the read multi-alignment and unsatisfied mate-pairs. In some cases, misplacing repetitive reads is benign and the resulting consensus sequence is correct. However, *amosvalidate *identifies the SNPs and unsatisfied mates as a signature of mis-assembly and reports the region as suspicious. We argue that this is the correct behavior, and for the false-positives we manually investigated, this was indeed the case. This is also the reason for such a large fraction of some assemblies being marked as suspicious (as high as 50% in some cases; Table 2). Acceptable specificity of our method is evidenced by the previous *D. virilis *example, where analysis of the 556 Kbp Celera Assembler contig revealed 6 suspicious regions that covered only 4% of the total sequence.

As would be expected, the wide variance of mis-assemblies found in the Phrap assemblies roughly correlates with genome repeat content, with no mis-assemblies being found in the small, non-repetitive assembly of *Neorickettsia sennetsu*, and 151 being found in the complex assembly of *Xanthomonas oryzae*, which contains many highly repetitive insertion sequence elements. The quality of these two assemblies is clearly reflected in the percentage of the genome marked as suspicious (3.5% and 55.1%, respectively). Also interesting are the three mis-assemblies identified in the *Mycoplasma capricolum *assembly, none of which were identified by our methods. Manual inspection of the reference alignment shows tandem repeat expansions of lengths 42, 240, and 654 bp. However, the assembly appears sound at these points with no fluctuation in CE statistic, good coverage, and few unsatisfied mates. Closure teams generally spend extra effort to properly handle repetitive regions, but if these repeats went unidentified during the closure process, it is possible that the reference sequence was mis-assembled. Unfortunately, the original assembly is not available for this genome, and only experimental validation could confirm the exact length and copy number of these repeats.

## Discussion

Due to the high cost of genome finishing, an increasing number of genomes, both prokaryotic and eukaryotic, are sequenced to only a draft level. Efforts at providing quality standards for draft genomes (for example, the comparative-grade standard [46]) have not yet addressed the issue of large-scale mis-assemblies, leading to the likely possibility that such mis-assemblies are present in the data deposited (at an ever increasing rate) in public databases. In addition, we have shown that mis-assemblies can persist even in 'finished' genomes. This situation is particularly troubling as scientists move away from the 'gene by gene' paradigm and attempt to understand the global organization of genomes. Without a clear understanding of the errors present in the data, such studies may draw incorrect conclusions. The validation tools described in this paper provide a first step towards a robust set of measures of assembly quality that go beyond the simple base-level measures commonly used. In future work, we will explore methods for converting mis-assembly features into a new type of assembly quality score representing the probability of mis-assembly at any location. The tools presented here, combined with tools designed to correct assemblies, will ultimately lead to automated finishing protocols that could dramatically improve the quality of draft-level assemblies.

In addition, we would like to stress the fact that the large-scale validation of assemblies cannot proceed without the availability of detailed information on the placement of individual reads within an assembly. Even if the raw reads are provided in the NCBI Trace Archive (as is the case for most current sequencing projects), mapping these reads to the assemblies deposited in public databases is a laborious, and error-prone process. Thus, we encourage the sequencing centers to release the details of their assemblies by submitting the complete assembly information to the NCBI Assembly Archive [37]. This community resource not only enables the application of high-throughput validation techniques, but also provides scientists with an interface for the manual inspection of assemblies.

## Abbreviations

BAC, bacterial artificial chromosome; CAF1, Comparative Analysis Freeze 1; CE statistic, compression-expansion statistic; SNP, single nucleotide polymorphism.

## Authors' contributions

AMP, MCS, and MP contributed to all portions of this work.

## Additional data files

The following additional data are available. Additional data file 1 contains a table listing the NCBI Taxonomy and RefSeq identifiers for the 16 genomes described in the Results section.

## Supplementary Material

Additional data file 1 NCBI Taxonomy and RefSeq identifiers for the 16 genomes described in the Results section. Click here for file
