# Supplementary material for: Genome assembly forensics: finding the elusive mis-assembly
Source: Genome Biol. 2008 Mar 14;9(3):R55. doi: 10.1186/gb-2008-9-3-r55 (PMC2397507; doi:10.1186/gb-2008-9-3-r55)
Supplement: Additional data file 1 — NCBI Taxonomy and RefSeq identifiers for the 16 genomes described in the Results section. [file gb-2008-9-3-r55-S1.pdf]

Table S1. NCBI Taxonomy and RefSeq identifiers for the 16 genomes described in the Results section. All 16 genomes and corresponding sequencing reads were obtained from The Institute for Genomic Research or the J. Craig Venter Institute. All sequencing data is also available from the NCBI Trace Archive. Three finished genomes have not been released as of publication and are marked with 'NA' in the RefSeq column. Multiple RefSeq IDs are listed for genomes with multiple chromosomes and/or plasmids.

Genome	Taxonomy ID	RefSeq ID
Bacillus anthracis Ames Ancestor	261594	NC_007530<br>NC_007323<br>NC_007322
Brucella suis 1330	204722	NC_004310<br>NC_004311
Campylobacter jejuni RM1221	195099	NC_003912
Chlamydophila caviae GPIC	227941	NC_003361<br>NC_004720
Coxiella burnetii RSA 493	227377	NC_002971<br>NC_004704
Dehalococcoides ethenogenes 195	243164	NC_002936
Fibrobacter succinogenes S85	59374	NA
Listeria monocytogenes 4b F2365	265669	NC_002973
Mycoplasma capricolum ATCC 27343	340047	NC_007633
Neorickettsia sennetsu Miyayama	222891	NC_007798
Prevotella intermedia 17	246198	NA
Pseudomonas syringae pv. tomato str. DC3000	223283	NC_004578<br>NC_004632<br>NC_004633
Staphylococcus aureus subsp. aureus COL	93062	NC_002951<br>NC_006629
Streptococcus agalactiae 2603V/R	208435	NC_004116
Wolbachia pipientis wMel	163164	NC_002978
Xanthomonas oryzae pv. oryzae PX099A	360094	NA
